# Supplementary material for: WOX5 Suppresses CYCLIN D Activity to Establish Quiescence at the Center of the Root Stem Cell Niche
Source: Curr Biol. 2014 Aug 18;24(16):1939–44. doi: 10.1016/j.cub.2014.07.019 (PMC4148176; doi:10.1016/j.cub.2014.07.019)
Supplement: Document S1. Supplemental Experimental Procedures, Figures S1–S3, and Table S1 [file mmc1.pdf]

Current Biology, Volume *24*

Supplemental Information

WOX5 Suppresses *CYCLIN D* Activity to Establish Quiescence at the Center of the Root Stem Cell Niche

Celine Forzani, Ernst Aichinger, Emily Sornay, Viola Willemsen, Thomas Laux, Walter Dewitte, and James A.H. Murray

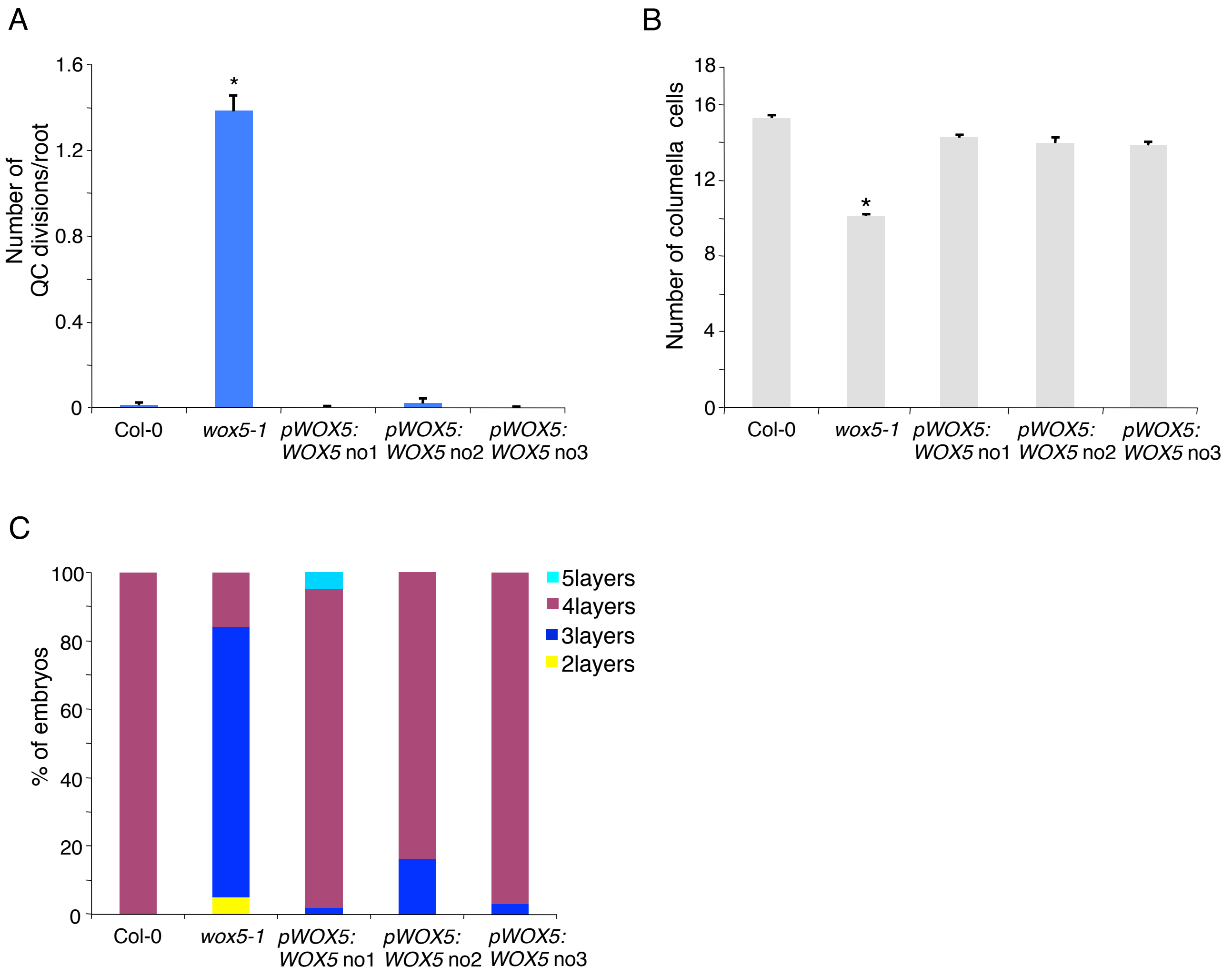

Figure S1. *wox5-1* complementation with a genomic WOX5 construct.

Related to figure 1.

Two additional *wox5-1* mutant plant lines expressing a WOX5 genomic DNA fragment under its own promoter *pWOX5:WOX5* no2 and no3 show rescue of *wox5-1* columella and QC defects, similar to the line *pWOX5:WOX5* no1 presented in Figure 1.

(A) Quantification of the number of transverse divisions occurring in the QC of mature embryos. (B) Quantification of the number of cells in the embryonic columella. (C) Quantification of the number of layers in the embryonic columella. (A-C) The number of embryos examined was: WT Col-0 (n=73), *wox5-1* (n=91), *pWOX5:WOX5* no1 (n=45), *pWOX5:WOX5* no2 (n=40), *pWOX5:WOX5* no3 (n=40). Error bars represent standard Errors. Student's t test, *p<0.00001

QC: quiescent centre

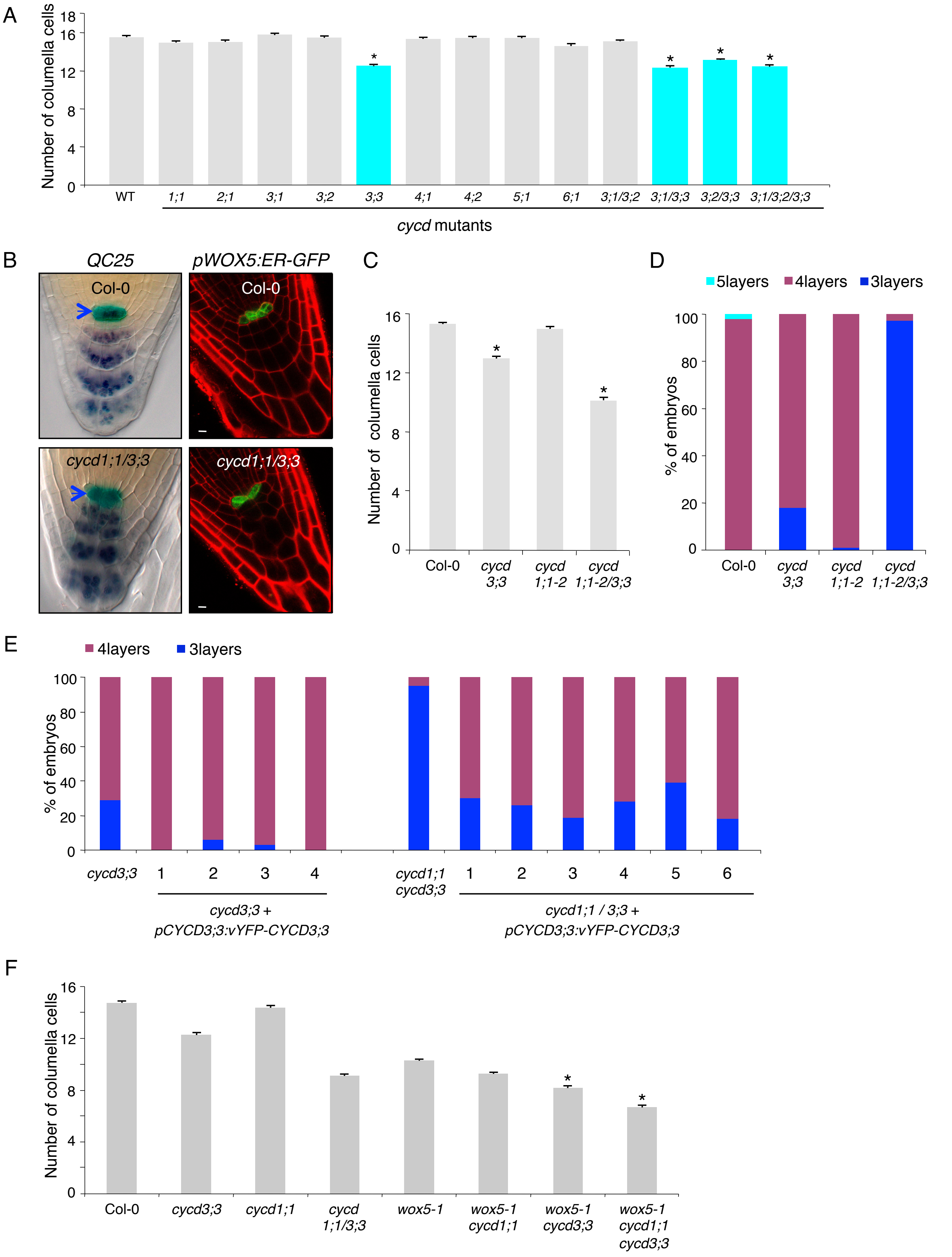
A
Number of columella cells
WT
1;1
2;1
3;1
3;2
3;3
4;1
4;2
5;1
6;1
3;1/3;2
3;1/3;3
3;2/3;3
3;1/3;2/3;3
cycd mutants
B
QC25
pWOX5:ER-GFP
Col-0
Col-0
cycd1;1/3;3
cycd1;1/3;3
C
Number of columella cells
Col-0
cycd 3;3
cycd 1;1-2
cycd 1;1-2/3;3
D
5layers
4layers
3layers
% of embryos
Col-0
cycd 3;3
cycd 1;1-2
cycd 1;1-2/3;3
E
4layers
3layers
% of embryos
cycd3;3
1
2
3
4
cycd3;3 + pCYCD3;3:vYFP-CYCD3;3
cycd1;1 cycd3;3
1
2
3
4
5
6
cycd1;1 / 3;3 + pCYCD3;3:vYFP-CYCD3;3
F
Number of columella cells
Col-0
cycd3;3
cycd1;1
cycd 1;1/3;3
wox5-1
wox5-1 cycd1;1
wox5-1 cycd3;3
wox5-1 cycd1;1 cycd3;3

Figure S2. *CYCD3;3*, *CYCD1;1* and *WOX5* regulate cell division in the embryonic columella.

Related to figure2.

(A) Quantification of the number of columella cells in the mature embryo. Approximately 30 embryos were counted for each genotype. *cycd3;3* mutants show less columella cells in the embryonic root compared to the WT Col-0 or to other *cycd* mutants. (B) *QC25* and *pWOX5:ER-GFP* expression in WT Col-0 and *cycd1;1 cycd3;3* 5-day-old seedlings. The enhancer trap line *QC25* expresses the ß-glucuronidase (GUS activity indicated by the blue arrow) in the QC. Starch granules in amyloplasts were stained dark blue by lugol (left panel). Cell walls were counterstained by propidium iodide (right panel). Scale bars= 5 μm. (C-D) A second loss-of-function allele of *CYCD1;1, cycd1;1-2* in combination with *cycd3;3* recapitulates the phenotypic abnormalities observed in *cycd1;1 cycd3;3* mutant plants. Approximately 50 embryos were counted for each genotype. (C) Quantification of the number of columella cells in the mature embryo. (D) Quantification of the number of layers in the embryonic columella. (E) A CYCD3;3 genomic DNA fragment tagged with vYFP was expressed under the control of the *CYCD3;3* promoter in *cycd3;3* or *cycd1;1 cycd3;3* mutants. Four different transgenic lines show complementation of the *cycd3;3* mutant defects. Six different transgenic lines show complementation of the *cycd1;1 cycd3;3* mutant defects. Quantification of the number of layers in the embryonic columella. Approximately 30 embryos were counted for each genotype. (F) A reduced number of columella cells was observed in *wox5 cycd1;1 cycd3;3* embryos compared to *wox5-1* or *cycd1;1 cycd3;3* embryos. Quantification of the number of columella cells in the mature embryo. The number of embryos examined was WT Col-0 (n=108), *cycd3;3* (n=101), *cycd1;1* (n=102), *cycd1;1 cycd3;3* (n=119), *wox5-1* (n=107), *wox5 cycd1;1* (n=118), *wox5 cycd3;3* (n=109), *wox5 cycd1;1 cycd3;3* (n=116).

(A, C and F) Error bars represent standard errors. Student t test *<0.00001. (F) * indicates a statistical difference between *cycd1;1 cycd3;3* and the different mutant combinations.

QC: quiescent centre

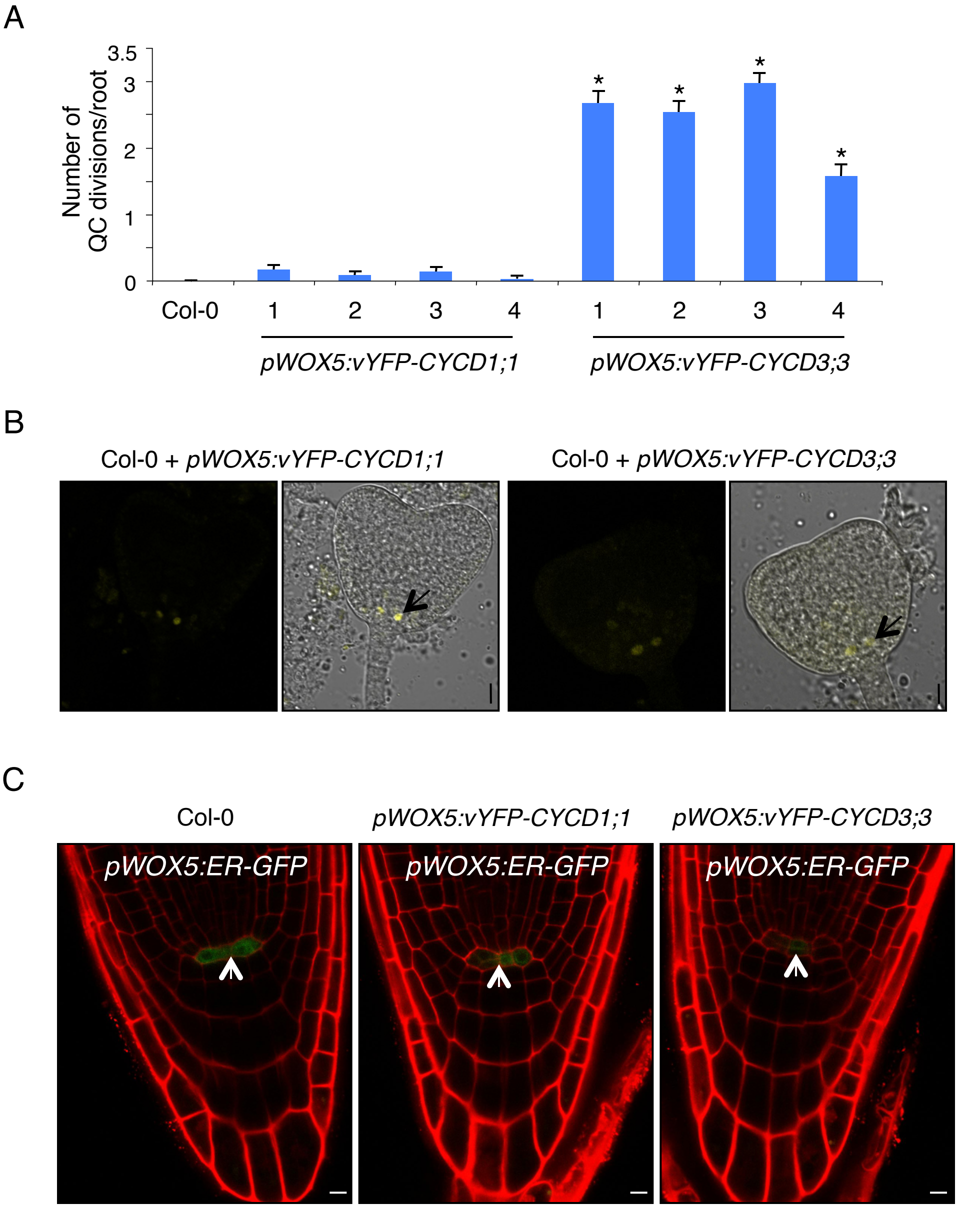

Figure S3. Increasing *CYCD* expression in the QC is sufficient to induce cell division in the QC.

Related to figure 3.

(A-C) A CYCD1;1 or a CYCD3;3 genomic DNA fragment tagged with vYF*P* was expressed under the control of the *WOX5* promoter in WT Col-0 plants. (A) Quantification of the number of transverse divisions occurring in the QC of mature embryos. Approximately 30 embryos were counted for each genotype. Error bars represent standard errors. Student t test *<0.00001 (B) The subcellular localization of *pWOX5:vYFP-CYCD1;1* or *pWOX5:vYFP-CYCD3;3* fusion proteins was examined in WT Col-0 heart stage embryos. The CYCD fusion proteins were detected in the nuclei (indicated by the black arrow).

The left panel shows the vYFP fluorescence and the right panel the vYFP fluorescence merged with the differential interference contrast image (DIC). Scale bars, 10 μm. (C) Five-day-old seedlings showing *pWOX5:ER-GFP* expression in the QC of WT Col-0 and *pWOX5:vYFP-CYCD1;1* or *pWOX5:vYFP-CYCD3;3* expressed in WT Col-0. Cell walls were counterstained by propidium iodide. F1 populations were used with *pWOX5:ER-GFP* being hemizygous in all the genotypes. White arrows indicate the QC. Scale bars= 5 μm.

Supplemental Table

Table S1. Quantification of the number of CSC layers in *cycds* and *wox5* mutant combinations.

Related to figure 2.

	**0 CSC (%)**	**1 CSC (%)**	**2 CSC (%)**	**N**
Col-0	0	84	16	31
cycd3;3	6	94	0	31
cycd1;1	3	58	39	31
cycd1;1 cycd3;3	32	68	0	32
wox5-1	100	0	0	31
wox5-1 cycd1;1	97	3	0	32
wox5-1 cycd3;3	100	0	0	31
wox5-1 cycd1;1 cycd3;3	94	6	0	32

3-day-old seedlings were visualized by propidium iodide. The percentage of roots with 0, 1 or 2 layers of CSC were counted. The graph in figure 2O scored these seedlings for their number of transverse divisions occurring in the QC.

Supplemental Experimental procedures

Plant growth conditions

Seeds were germinated on root medium (2.2 g L^{-1} Murashige Skoog, 0.75 % sucrose and 1.5 % agar pH 5.8) or GM medium (4.4 g L^{-1} Murashige Skoog, 1.5 % sucrose, 0.5 g L^{-1} 2-(N-Morpholino) ethanesulfonic acid sodium salt (MES) and 1 % agar, pH 5.8). The seeds were stratified at 4°C for 48 h and were then transferred to 22°C under long day conditions (16 h light, 8 h dark) for germination and growth.

Plant lines and constructs

The *Arabidopsis thaliana* ecotype Columbia 0 was used for all the experiments. The *cycd3;1*, *cycd3;2*, *cycd3;3* [S1], *cycd1;1* [S2], *wox5-1*, *wox5-3* [S3] mutants, *QC25, QC46* [S4] and *pWOX5:ER-GFP* [S5] lines have been described previously. The loss-of-function mutant line *cycd1;1-2* (SM_3.20203) was obtained from the Nottingham Arabidopsis stock centre. It has a T-DNA insertion in the 2nd intron of the *CYCD1;1* gene. The site of insertion sequenced 3' from the T-DNA is: ATGATTATATTAATCAGCTTTAATTTCTGTAAAATAGGAAACGAGTGGTTG GCCAATGCAACTTTT. The primers used for genotying are: T-DNA: ACCGTCGACTACCTTTTTTCTTGTAGTG, CYCD1;1 forward: CTTTACGCTCGTCGATTACCG and CTCD1;1 reverse: GCATTGGCCAACCACTCGTTTC. The absence of the *CYCD1;1* transcript in this line was confirmed by RT-PCR with primers spanning the T-DNA (data not shown). Plants were transformed using the floral dipping method [S6]. Primary transformants were selected on GM medium containing kanamycin (50 µg/ml) or DL-phosphinothricin (12 µg/ml).

The *CYCD1;1* and *CYCD3;3* promoter sequence was amplified by PCR from total genomic DNA derived from Col-0 seedlings. *pCYCD1;1* (5665 bp upstream of the transcriptional start site (TSS)) and *pCYCD3;3* (4805 bp upstream of the TSS) were introduced into the gateway vector pENTR/D-TOPO (Invitrogen) with a SmaI site introduced at the 5' end of the promoter. These constructs were then recombined into the binary vector pKGWFS7 [S7] generating *pCYCD1;1:eGFP-GUS* and

pCYCD3;3:eGFP-GUS.

pWOX5:vYFP-CYCD1;1, *pWOX5:vYFP-CYCD3;3* and *pCYCD3;3:vYFP-CYCD3;3* constructs were expressed in plants from the binary vector pGreenII 0229 [S8]. The pGreenII 0229 vector was modified to be able to make these constructs. First, the *35S* promoter was cloned ApaI/XhoI into the pGreenII 0229. Additional restriction sites in the *p35S* primers created new restriction sites in the pGreenII 0229 vector: ApaI-PmeI/*p35S*/MluI-XhoI. The vYFP tag was cloned behind the *35S* promoter MluI/XhoI. Additional restriction sites in the vYFP primers created: MluI/vYFP/NotI-AscI-XhoI. The *CYCD3;3* (4805 bp upstream of the TSS) or the *WOX5* (4658 bp upstream of the TSS) promoters were cloned SmaI/AscI into this modified pGreenII 0229 by replacing the *35S* promoter PmeI/MluI. The genomic DNA sequence of CYCD1;1 or of CYCD3;3 including the 3' UTR was amplified by PCR from total genomic DNA derived from Col-0 seedlings. Each fragment was cloned in frame to the vYFP N-terminal tag NotI/AscI behind the *WOX5* promoter. In addition, the CYCD3;3 gDNA fragment was cloned in frame to the vYFP N-terminal tag NotI/AscI behind the *CYCD3;3* promoter.

The *pWOX5:WOX5* construct was expressed in the *wox5 QC184* mutant background from the binary vector pGreenII. A WOX5 genomic fragment was amplified using the primers: CCCGGGTGGTAGCGAACTAGGAATTGTATGTGC and GGCGCGCCTCCGACGGAACTTGAGTTTGCTTC and cloned AscI/XmaI into pGreenII.

Histochemical staining

5-day-old seedlings were fixed in 90 % acetone for 30 min at 4°C, washed three times with 50 mM sodium phosphate buffer (pH 7.0) and subsequently stained for up to 16 hours at 37°C, in 50 mM sodium phosphate buffer (pH 7.0), 10 mM $K_3Fe(CN_6)$, 10 mM $K_4Fe(CN_6)$ containing 1 mM 5-bromo-4-chloro-3-indolyl-ß-D-glucuronide. Subsequently, seedlings were incubated in Lugol's solution (SIGMA) for 30 seconds and rinsed with water. Root tips were mounted in chloral hydrate (2.7 g chloral hydrate in 1 ml water) and were visualized with a Leica MZ16FA stereomicroscope using differential interference contrast optics.

Confocal Microscopy

Dry seeds were imbibed in water O/N. Mature embryos were popped out from the seed and stained in fixative (50 % ethanol / 10 % acetic acid) for 24 h. Embryos were rinsed with water and incubated in 1 % periodic acid for 40 min. at room temperature. Embryos were rinsed again and then stained with a Schiff reagent (100 mM sodium metabisulphite, 0.15N HCL, propidium iodide 100 µg/ml) for 1 h at room temperature. Embryos were rinsed with water and cleared with a chloral hydrate solution (2.7 g chloral hydrate in 1 ml water). Embryos were then mounted on a slide. Roots from 5-day-old seedlings were directly mounted in propidium iodide solution (40 µg/ml) to stain the cell wall. Samples were visualized by confocal microscopy Zeiss LSM 710.

Chromatin Immunopreciptiation

The ChIP was carried out using an anti-WOX5 antibody to co-immunoprecipitate the DNA interacting with a WOX5-GR fusion protein expressed from the *35S* promoter in the WT Col-0 background. Roots of 7-day-old seedlings, treated with 5 µM dexamethasone and 0.15 % silwet were used for ChIP experiments according to Kwon *et al.* [S9] with some minor modifications. 1 to 1.2 g of root tissue was used per replicate. Lysed nuclei were sonicated with a Bioruptor sonicator (Diagenode) to a fragment size of 300 to 600 bp. Pre-clearing was performed with a 1:1 mixture of Protein-A/Protein-G dynabeads (Invitrogen) for 1.5 h. The DNA was precipitated by using an anti-WOX5 antibody at 4°C for 16 hours followed by incubation with a 1:1 mixture of Protein-A/Protein-G dynabeads for 2 hours. Cross-linking was reversed by incubation of the beads in 10 % chelex at 95°C for 10 min, followed by incubation with 20 µg proteinase K at 50°C for 30 min and at 95°C for 10 min. The supernatant was collected and combined with one washing step of the chelex/dynabeads with TE buffer. 3 µl per well was used for qPCR using SYBR Green II (Roche).

List of primers used to amplify the different promoter regions:

CYCD3;3-1750:

CTAATGGAACATATTGTAGACCTATTTGG

AACAAAGAAATTCCTTATCATATCGCT

CYCD3;3 -700:

ACACAATGACAGTGACTCTACACATTACG

CGTCATTAGTACTTTGATTTTGGTTAATACAAC

CYCD3;3_5UTR

AGAGGACAAGCGTGAAATAAAACCCT

CTCTGGATTCTTCACTCTGTGTGAGA

CYCD3;3_intron

GATCTCTTGAATCTACTTGATTTTGTTGT

GGCTAACACAGAAGGACTAAAACTCAG

CYCD3;3_3UTR

TGTCAACAACCCATCAAAATGTGT

TCCATAAACACAAGAACCGAGAACCA

CYCD3;1-400

TTGTGTGTTCATCATCCTCTTGAG

GCCGATAAGTTCCGTTTCTTAAAG

CYCD6;1-1000

TAACTGTTTGGACGAGATTCCAAAGT

GTTGTCATTTACTCATAAATAGAGCTGATG

CYCD6;1-100

ACCATCTCTCTCTATCTCACAATAAAGAC

TTCTTGAATTGGTGTTAGAAGATGAAGA

eIF4

CACGCCCTGGAGTTCCAACAAC

GCAAATTGAGAAGGTCATGAGG

Supplemental References

S1. Dewitte, W., Scofield, S., Alcasabas, A.A., Maughan, S.C., Menges, M., Braun, N., Collins, C., Nieuwland, J., Prinsen, E., Sundaresan, V., *et al.* (2007). Arabidopsis CYCD3 D-type cyclins link cell proliferation and endocycles and are rate-limiting for cytokinin responses. Proc. Natl. Acad.

Sci. USA *104*, 14537-14542.

S2. Masubelele, N.H., Dewitte, W., Menges, M., Maughan, S., Collins, C., Huntley, R., Nieuwland, J., Scofield, S., and Murray, J.A. (2005). D-type cyclins activate division in the root apex to promote seed germination in Arabidopsis. Proc. Natl. Acad. Sci. USA *102*, 15694-15699.

S3. Sarkar, A.K., Luijten, M., Miyashima, S., Lenhard, M., Hashimoto, T., Nakajima, K., Scheres, B., Heidstra, R., and Laux, T. (2007). Conserved factors regulate signalling in Arabidopsis thaliana shoot and root stem cell organizers. Nature *446*, 811-814.

S4. Sabatini, S., Heidstra, R., Wildwater, M., and Scheres, B. (2003). SCARECROW is involved in positioning the stem cell niche in the Arabidopsis root meristem. Genes Dev. *17*, 354-358.

S5. ten Hove, C.A., Willemsen, V., de Vries, W.J., van Dijken, A., Scheres, B., and Heidstra, R. (2010). SCHIZORIZA encodes a nuclear factor regulating asymmetry of stem cell divisions in the Arabidopsis root. Curr. Biol. *20*, 452-457.

S6. Clough, S.J., and Bent, A.F. (1998). Floral dip: a simplified method for Agrobacterium-mediated transformation of Arabidopsis thaliana. Plant J. *16*, 735-743.

S7. Karimi, M., Inze, D., and Depicker, A. (2002). GATEWAY vectors for Agrobacterium-mediated plant transformation. Trends Plant Sci. *7*, 193-195.

S8. Hellens, R.P., Edwards, E.A., Leyland, N.R., Bean, S., and Mullineaux, P.M. (2000). pGreen: a versatile and flexible binary Ti vector for Agrobacterium-mediated plant transformation. Plant Mol. Biol. *42*, 819-832.

S9. Kwon, C.S., Chen, C., and Wagner, D. (2005). WUSCHEL is a primary target for transcriptional regulation by SPLAYED in dynamic control of stem cell fate in Arabidopsis. Genes Dev. *19*, 992-1003.
