# Supplementary material for: WOX5 Suppresses CYCLIN D Activity to Establish Quiescence at the Center of the Root Stem Cell Niche
Source: Curr Biol. 2014 Aug 18;24(16):1939–44. doi: 10.1016/j.cub.2014.07.019 (PMC4148176; doi:10.1016/j.cub.2014.07.019)
Supplement: Document S2. Article plus Supplemental Information [file mmc2.pdf]

Current Biology 24, 1939–1944, August 18, 2014 ©2014 The Authors http://dx.doi.org/10.1016/j.cub.2014.07.019

Report

WOX5 Suppresses *CYCLIN D* Activity to Establish Quiescence at the Center of the Root Stem Cell Niche

Celine Forzani,[1] Ernst Aichinger,[2] Emily Sornay,[1] Viola Willemsen,[3] Thomas Laux,[2] Walter Dewitte,[1,*] and James A.H. Murray[1,*]
[1]School of Biosciences, Cardiff University, Museum Avenue, Cardiff CF10 3AX, Wales, UK
[2]Faculty of Biology, BIOSS Centre for Biological Signalling Studies, Albert-Ludwigs-University Freiburg, 79104 Freiburg, Germany
[3]Plant Developmental Biology, Wageningen University and Research Centre, Droevendaalsesteeg 1, 6708 PB Wageningen, The Netherlands

Summary

In *Arabidopsis*, stem cells maintain the provision of new cells for root growth. They surround a group of slowly dividing cells named the quiescent center (QC), and, together, they form the stem cell niche (SCN). The QC acts as the signaling center of the SCN, repressing differentiation of the surrounding stem cells [1] and providing a pool of cells able to replace damaged stem cells [2, 3]. Maintenance of the stem cells depends on the transcription factor WUSCHEL-RELATED HOMEOBOX 5 (WOX5), which is specifically expressed in the QC [4]. However, the molecular mechanisms by which WOX5 promotes stem cell fate and whether WOX5 regulates proliferation of the QC are unknown. Here, we reveal a new role for WOX5 in restraining cell division in the cells of the QC, thereby establishing quiescence. In contrast, WOX5 and CYCD3;3/CYCD1;1 both promote cell proliferation in the nascent columella. The additional QC divisions occurring in *wox5* mutants are suppressed in mutant combinations with the D type cyclins *CYCD3;3* and *CYCD1;1*. Moreover, ectopic expression of *CYCD3;3* in the QC is sufficient to induce cell division in the QC. WOX5 thus suppresses QC divisions that are otherwise promoted by CYCD3;3 and CYCD1;1, in part by interacting with the *CYCD3;3* promoter to repress *CYCD3;3* expression in the QC. Therefore, we propose a specific role for WOX5 in initiating and maintaining quiescence of the QC by excluding *CYCD* activity from the QC.

Results and Discussion

WOX5 Stimulates Columella Development and Initiates Quiescence in the Embryonic QC

The root apical meristem (RAM) is initiated during embryogenesis when the uppermost suspensor cell or hypophysis divides asymmetrically to produce an upper lens-shaped cell, the progenitor of the quiescent center (QC), and a larger basal cell that gives rise to the columella stem cells (CSCs) and columella [5]. The QC maintains a low frequency of division, which reduces vulnerability to DNA damage [2, 3], and also specifies adjacent cells to become the stem cells that actively divide to renew themselves [6, 7]. How this quiescence is initiated and maintained in the midst of proliferating stem cells is unknown. Because *WUSCHEL-RELATED HOMEOBOX 5* (*WOX5*) expression is first detected in the hypophysis and remains specifically expressed in the QC during both embryogenesis and postgermination growth [4, 8], we tested whether WOX5 regulates cell division in the descendants of the hypophysis.

We used mature embryos as a "read out" of embryonic development in which cell numbers and patterns are easily studied. WOX5 is required postembryonically to maintain CSCs in the RAM [4], so we reasoned it might also be active during embryogenesis when the columella is forming. Therefore, we counted the number of columella cells (CCs) distal to the QC and the number of columella layers in two loss-of-function alleles of *WOX5* (Figures 1A–1C). The number of columella layers was reduced in *wox5* embryos, with 80% showing three layers of CCs instead of four in wild-type (WT) Col-0 (Figures 1E and 1F). Occasionally, enlarged CCs spanning two layers were observed in 92% of *wox5* mutant roots (Figures 1B and 1C). Introducing a construct carrying the *WOX5* locus into *wox5-1* mutants completely restored the number of columella layers (Figures 1D–1F; Figures S1B and S1C available online), confirming that the observed defects were caused by the *wox5-1* mutation.

This led us to conclude that *WOX5* functions in embryogenesis to ensure the correct number of cells in the columella. Because *WOX5* is expressed specifically in the embryonic QC, we tested whether loss of *WOX5* also affected the cellular organization of the QC and specifically the initiation of quiescence. QC cell divisions were quantified by determining the number of transverse divisions (Figures 1B and 1C). *wox5* embryonic roots showed an increase in QC cell divisions, with on average 1.4 divisions occurring per root compared to 0.013 divisions in WT Col-0 (Figure 1G), and this was suppressed in *wox5-1* mutants expressing a *pWOX5:WOX5* construct (Figures 1D, 1G, and S1A). Furthermore, the increase in QC cell division was maintained after germination in the primary root (Figure 1G).

These results demonstrate that in addition to its previously documented role in maintaining CSCs postgermination [4], *WOX5* mediates cell numbers in the nascent columella and cell autonomously restricts cell division in the QC.

CYCD1;1 and *CYCD3;3* Control Cell Division during the Morphogenesis of the Columella and Prevent Premature Stem Cell Differentiation

The molecular mechanisms regulating cell division in the developing root pole are currently unknown. The mitotic cell cycle is driven by the action of cyclin-dependent kinase (CDK)/cyclin complexes, and CDKA/cyclin D (CYCD) complexes control the commitment point at the G_1/S transition through reversible phosphorylation of the RETINOBLASTOMA-RELATED (RBR) protein [9–12]. RBR has recently been shown to act cell autonomously in the QC to limit cell divisions [2, 13], and it plays a key role in restraining cell proliferation in root stem cells [14]. However, RBR is encoded by a single gene expressed throughout

*Correspondence: dewittew@cardiff.ac.uk (W.D.), murrayja1@cardiff.ac.uk (J.A.H.M.)
This is an open access article under the CC BY license (http://creativecommons.org/licenses/by/3.0/).

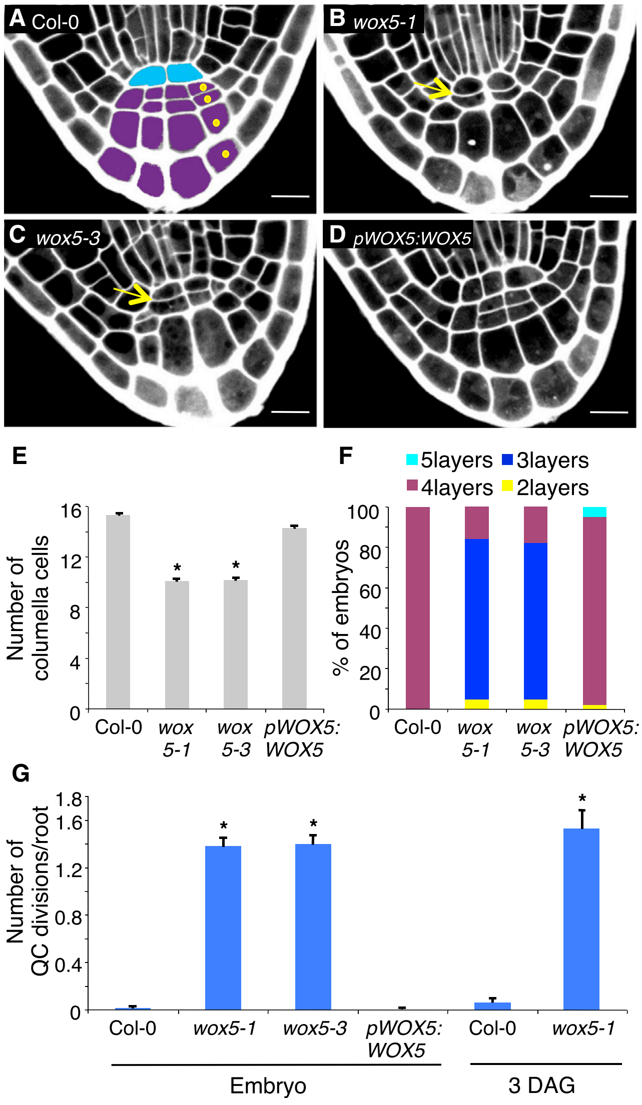

Figure 1. During Embryogenesis, *WOX5* Suppresses Cell Division in the QC and Induces Divisions in the Columella

(A–D) Cell walls in mature embryos were visualized by propidium iodide staining. *wox5* mutants display transverse divisions in the QC, as indicated by the yellow arrow, and a reduced number of CCs compared to WT Col-0. Scale bars represent 10 μm.

(A) Mature embryo with the different cell types color coded: QC in blue; columella in purple.

(D) The WOX5 genomic DNA was expressed from the *WOX5* promoter in *wox5-1*.

(E) Quantification of the number of cells in the embryonic root. The number of cells in the columella was counted according to the scheme in (A).

(F) Quantification of the number of layers in the columella. The maximum number of layers was counted (see yellow dots in A).

(G) Quantification of the number of transverse divisions occurring in the QC of mature embryos or 3-day-old roots.

(E–G) Numbers of embryos examined: WT Col-0, n = 73; *wox5-1*, n = 91; *wox5-3*, n = 85; *pWOX5:WOX5*, n = 45. Thirty seedlings were counted for the WT Col-0 or for the mutant *wox5-1*. Error bars show SEs (E and G). Student's t test, *p < 0. 00001.

QC, quiescent center; DAG, days after germination. See also Figure S1.

the root [14]. Because the *Arabidopsis* genome contains ten *CYCD* genes with distinct patterns of expression in the root [15, 16], these are more likely candidates to provide tissue-specific regulation of RBR activity. Furthermore, elevating CYCD3;1 levels results in additional CSC layers [14], and *CYCDs* respond to intrinsic and extrinsic signals [17–19].

To investigate whether specific *CYCDs* control cell division in the developing root pole and stem cell niche, we screened mature embryos of single loss-of-function alleles of the various *CYCDs* for defects in cell division in the columella (Figure S2A). Only *cycd3;3* mutants showed a reduced number of CCs, with 25% of *cycd3;3* embryos having three columella layers instead of four (Figures 2A, 2B, and 2M). Because the *Arabidopsis CYCD3* subgroup has three members (*CYCD3;1*, *CYCD3;2*, *CYCD3;3*) [18], different combinations of the *cycd3* mutant alleles were assessed for enhanced defects with respect to cell division in the columella (Figure S2A). *cycd3;1-3* embryos lacking all *CYCD3* function were very similar to *cycd3;3* embryos, indicating that from the *CYCD3* subgroup, only *CYCD3;3* is essential for normal cell division activity in the embryonic columella.

Because redundancy with other *CYCD* genes might be responsible for the relatively weak *cycd3;3* mutant phenotype, we examined the tissue specificity of their expression [16] to identify other potential cofunctioning candidates. Monitoring the GFP fluorescence from *CYCD* promoters driving a GUS-GFP reporter construct showed that only *CYCD1;1* displayed specific expression in the embryonic QC, persisting from heart to torpedo stage (Figure 2I) [16]. In contrast, *CYCD3;3* expression was not observed in the QC but was apparent in the root cap from heart until torpedo stage (Figure 2I). Given that *WOX5* acts from the QC to maintain CSCs, we tested whether *CYCD1;1* might similarly influence the divisions in the columella from its site of expression in the QC. The *cycd1;1* mutants showed no detectable phenotype, but, in combination with *cycd3;3*, they did enhance the *cycd3;3* phenotype (Figures 2C and 2D). One hundred percent of *cycd1;1 cycd3;3* embryos displayed three columella layers instead of four (Figure 2M), and occasionally, enlarged CCs spanning two layers were observed in 56% of *cycd1;1 cycd3;3* mutant roots.

After germination, CCs are generated by the dividing CSCs at the distal side of the QC. Differentiated CCs accumulate amyloplasts, starch-accumulating plastids, which are absent from CSCs. *WOX5* is required to maintain the identity of the CSCs, which differentiate in *wox5* mutants [4].

Given that the embryonic columella phenotype of *cycd1;1 cycd3;3* mutants is similar to that of *wox5*, we assessed whether *CYCD1;1* and *CYCD3;3* are required to maintain CSCs in the primary root. One or two layers of CSCs are detected in 5-day-old WT roots (Figure 2J). However, in 35% of *cycd1;1 cycd3;3* roots, amyloplasts accumulated in CSCs, indicating a loss of CSC identity and premature differentiation into CCs (Figure 2J). *cycd3;3* mutant roots also showed early differentiation, albeit to a lesser extent than *cycd1;1 cycd3;3*.

Because QC function is essential to repress CSC differentiation, we tested whether QC identity was modified in *cycd1;1 cycd3;3* roots. Three different QC-specific molecular markers, *QC25* (Figure S2B), *QC46* (Figure 2K) [20], and *WOX5:ER-GFP* (Figure S2B) [21], were normally expressed in the QC of *cycd1;1 cycd3;3* mutant roots, suggesting that the QC identity is unaffected.

To confirm that the phenotypes observed in *cycd1;1 cycd3;3* mutants were caused by loss-of-function of these cyclins, we examined a second loss-of-function allele of *CYCD1;1*, *cycd1;1-2*, in combination with *cycd3;3*. The *cycd1;1-2 cycd3;3* double mutant recapitulated the original *cycd1;1 cycd3;3* phenotype (Figures S2C and S2D). In the absence of a second loss-of-function allele of *CYCD3;3*, we complemented

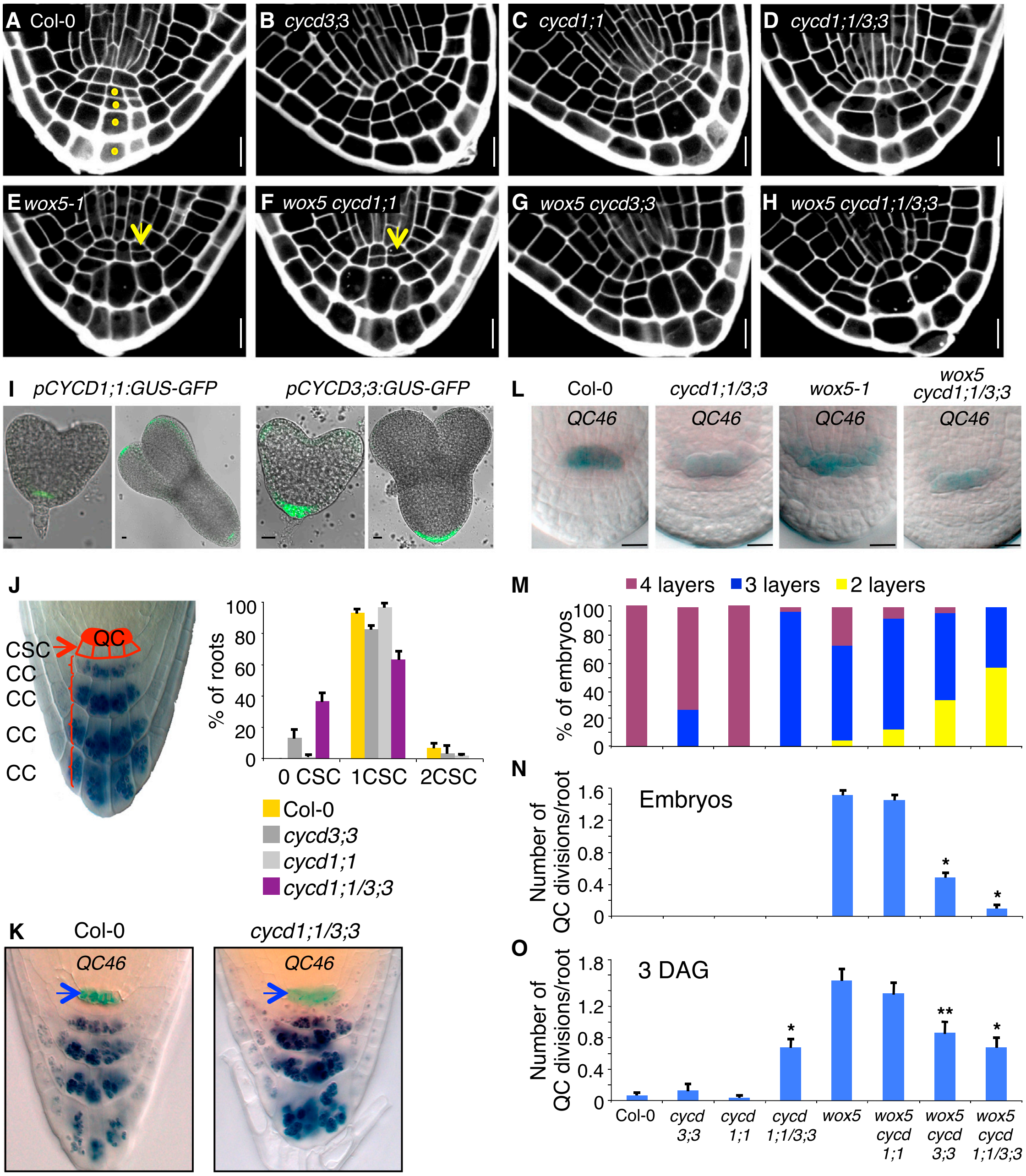

Figure 2. Genetic Interaction between *CYCD1;1/CYCD3;3* and *WOX5*

(A–H) Mature embryos were visualized by propidium iodide staining. Yellow arrows indicate transverse divisions occurring in the QC.

(A–D) *cycd;3;3* and *cycd1;1 cycd3;3* mutants show a reduced number of CCs in the embryonic root compared to WT Col-0.

(E–H) The transverse divisions occurring in the *wox5-1* QC are absent from the *wox5 cycd1;1 cycd3;3* QC.

(I) *pCYCD1;1:GUS-GFP* reporter line shows *GFP* expression in the embryonic QC. *pCYCD3;3:GUS-GFP* reporter line shows *GFP* expression in the embryonic root pole.

(J) Left: diagram illustrating the *Arabidopsis* root with the QC (colored in red), one layer of CSCs, and four layers of CCs. Right: percentage of roots with zero, one, or two layers of CSCs in 5-day-old roots. Error bars show SD from three biological repeats. Between 30 and 40 roots were examined for every genotype per experiment.

(K) *QC46* expression in WT Col-0 and *cycd1;1 cycd3;3* of 5-day-old seedlings. The enhancer trap line *QC46* expresses β-glucuronidase in the QC (GUS activity is indicated by the blue arrow). Starch granules in amyloplasts were stained dark blue by lugol.

(L) *QC46* expression in the QC of mature embryos.

(M) Quantification of the number of layers in the columella. The maximum number of layers was counted (see yellow dots in A).

(N) Quantification of the number of transverse divisions occurring in the QC of mature embryos (N) or 3-day-old roots (O).

(M and N) Numbers of embryos examined: Col-0, n = 108; *cycd3;3*, n = 101; *cycd1;1*, n = 102; *cycd1;1cycd3;3*, n = 119; *wox5-1*, n = 107; *wox5 cycd1;1*, n = 118; *wox5 cycd3;3*, n = 109; *wox5 cycd1;1 cycd3;3*, n = 116.

(O) Twenty-five seedlings were counted for each genotype.

Error bars show SEs (N and O). Student's t test, *p < 0.001, **p < 0.01. The asterisk (*) in (O) indicates a statistical difference between *wox5-1* and the different mutant combinations. Scale bars of (A)–(I) and (L) represent 10 μm. QC, quiescent center; DAG, days after germination. See also Figure S2.

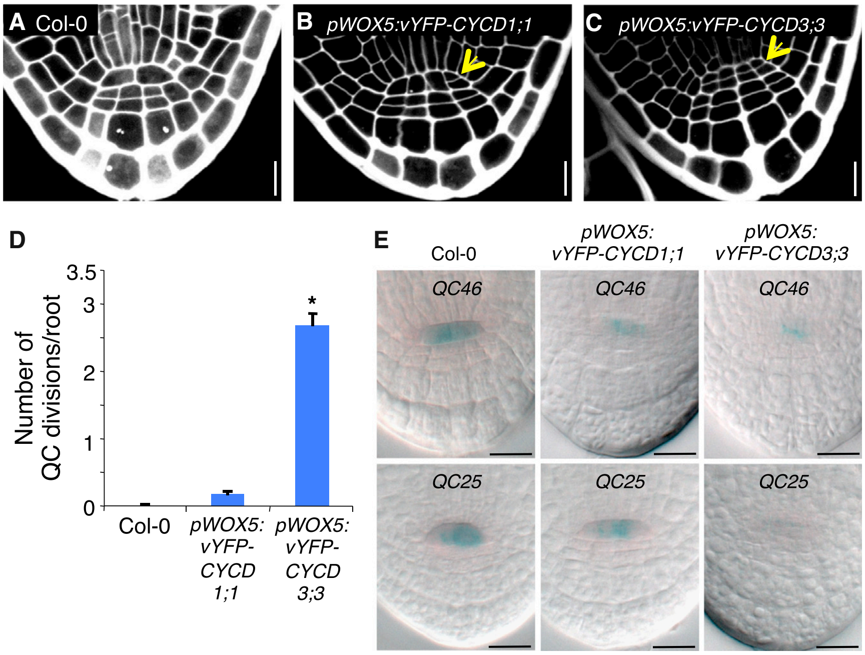

Figure 3. Ectopic *CYCD* Expression in the QC Is Sufficient to Induce QC Cell Division

(A–C) Mature embryos were visualized by propidium iodide staining. Yellow arrows indicate transverse divisions occurring in the QC of *pWOX5:vYFP-CYCD1;1* and *pWOX5:vYFP-CYCD3;3* expressed in WT Col-0. The CYCD1;1 or the CYCD3;3 genomic DNA was expressed from the *WOX5* promoter.

(D) Quantification of the number of transverse divisions occurring in the QC of mature embryos. Numbers of embryos examined: WT Col-0, n = 40; *pWOX5:vYFP-CYCD1;1*, n = 33; *pWOX5:-vYFP-CYCD3;3*, n = 34.

(E) *QC46* and *QC25* expression in the QC of mature embryos of WT Col-0 (reduced in Col-0 -expressing either *pWOX5:vYFP-CYCD1;1* or *pWOX5:vYFP-CYCD3;3*). F1 populations were used with *QC46* and *QC25*, being hemizygous in all the genotypes.

Scale bars of (A)–(C) and (E) represent 10 μm. See also Figure S3.

cycd3;3 and *cycd1;1 cycd3;3* double mutants with a construct encoding *vYFP* fused to a *CYCD3;3* genomic fragment under the control of the *CYCD3;3* promoter. This efficiently rescued both *cycd3;3* and *cycd1;1 cycd3;3* abnormalities (Figure S2E).

Taken together, we conclude that *CYCD1;1* and *CYCD3;3* stimulate cell division during the formation of the columella and prevent CSC differentiation to maintain the CSC niche postgermination.

WOX5 Acts through *CYCD1;1* and *CYCD3;3* to Control Cell Divisions in the QC

To assess whether *WOX5* might act through *CYCD1;1* and *CYCD3;3* to regulate cell division, we tested the genetic interaction of *wox5-1* with the *cycd1;1 cycd3;3* mutant. The additional QC cell divisions observed in 89% of *wox5-1* embryonic roots were almost totally suppressed in *wox5 cycd1;1 cycd3;3* (8%; Figures 2A–2H and 2N). This phenotype was already apparent in *wox5 cycd3;3* double mutant, in which only 45% of embryos showed divisions in the QC. Hence, both *CYCD1;1* and *CYCD3;3* contribute to the extra transverse divisions observed in the *wox5-1* embryonic QC. The identity of the QC was confirmed through expression of the QC-specific marker *QC46* [20]. *QC46* was normally expressed in the mature embryonic QC of *wox5-1 cycd1;1 cycd3;3* as it was in *wox5-1* or *cycd1;1 cycd3;3* roots (Figure 2L), indicating that QC development was unaffected.

We then tested whether *CYCD1;1* and *CYCD3;3* fulfil the same role postembryonically, being rate limiting for QC cell division in *wox5-1* mutant roots. Indeed, the additional QC cell divisions present in *wox5-1* mutants were reduced by 45% in *wox5 cycd3;3* and by 55% in *wox5 cycd1;1 cycd3;3* mutants (Figure 2O). However, we note that additional QC cell divisions were also seen in *cycd1;1 cycd3;3* roots. This would be consistent with the premature differentiation of the CSCs in the *cycd1;1 cycd3;3* roots (Figure 2J; Table S1), triggering additional cell divisions of the QC to replenish the CSC pool.

Taken together, these results suggest that *CYCD1;1* and *CYCD3;3* drive the abnormal divisions in the *wox5-1* QC.

In contrast, in the developing columella, *WOX5* seems to act, in part, independently from *CYCD3;3* and *CYCD1;1*. A further reduction in the number of CCs was observed in the triple *wox5-1 cycd1;1 cycd3;3* mutant compared to either *wox5-1* or *cycd1;1 cycd3;3*. Fifty-seven percent of *wox5-1 cycd1;1 cycd3;3* embryos had only two columella layers compared to 4% in *wox5-1* and 0% in *cycd1;1 cycd3;3* (Figures 2M and S2F). These results suggest that *WOX5* may partially act through *CYCD1;1/CYCD3;3* and also through additional unknown targets to control cell division in the embryonic columella.

Ectopic QC Expression of *CYCD3;3* Induces QC Cell Division

To further assess whether *CYCD* regulates cell division in the QC, we tested whether ectopic expression of *CYCD3;3* in the QC would be sufficient to induce cell division. Both the vYFP-CYCD3;3 and the vYFP-CYCD1;1 fusion proteins expressed under the control of the *WOX5* promoter triggered transverse divisions in the QC. Both proteins were consistently detected in the nucleus of the QC (Figure S3B), and vYFP-CYCD3;3 triggered QC divisions in 100% of embryos and vYFP-CYCD1;1 in 9% (Figures 3A–3D and S3A).

To analyze whether these additional divisions influenced QC identity, we examined the expression of the QC-specific markers, *QC46* and *QC25*, in the embryonic root (Figure 3E) and *pWOX5:ER-GFP* in the QC of 5-day-old roots (Figure S3C). Although activity of these QC-specific markers is apparent in WT Col-0 lines expressing *pWOX5:vYFP-CYCD1;1* or *pWOX5:vYFP-CYCD3;3*, all showed reduced expression, suggesting a potential partial loss of QC identity (Figures 3E and S3C).

These results suggest that restraining of CYCD activity in the QC is required to maintain both quiescence and the correct cellular identity in the QC.

CYCD3;3 Expression Is Suppressed by WOX5 in the QC

To establish whether *WOX5* acts to exclude *CYCD* expression from the QC, thereby maintaining its quiescence, we compared *CYCD1;1* or *CYCD3;3* expression in *wox5-1* and WT mutant roots by using a GUS-GFP fusion protein

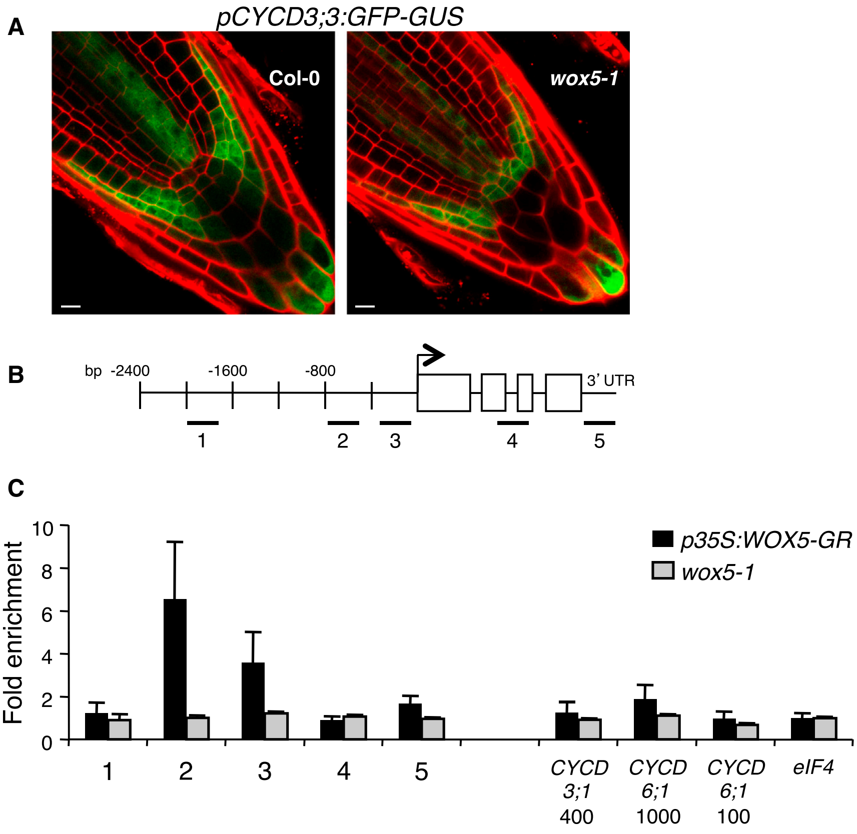

Figure 4. WOX5 Restricts *CYCD3;3* Activity from the QC and Binds to Its Promoter

(A) *pCYCD3;3:GUS-GFP* expression in *wox5-1* roots expands into the QC compared to WT Col-0 roots. Cell walls of 5-day-old roots were stained with propidium iodide. Scale bars represent 10 μm.

(B and C) ChIP experiments were performed with root samples from WT Col-0 plants expressing a *p35S:WOX5-GR* construct and *wox5-1* mutant plants. An anti-WOX5 antibody was used to immunoprecipitate WOX5, followed by qPCR of the *CYCD3;3* promoter and gene as illustrated in (B) and of the *CYCD3;1* and *CYCD6;1* promoter regions. The fold enrichment was calculated relative to the input and signal at the eukaryotic initiation factor (eIF4) gene. Error bars show SE from three technical replicates. The ChIP experiment was repeated three times, with similar results each time.

expressed under the control of the *CYCD* promoter. Differences were seen only for *CYCD3;3* expression, which was detected in the QC of *wox5-1* seedlings but was almost absent from WT QC (Figure 4A). In WT roots, *CYCD3;3* was also expressed in the distal columella layers, the lateral root cap, the epidermal stem cells, and the stele, but this pattern was unaltered in *wox5-1* mutant roots. This *CYCD3;3* promoter is functional in planta because *vYFP-CYCD3;3* expressed under its control complements the *cycd1;1 cycd3;3* mutant phenotype (Figure S2E). These results were reproducible with three independent *pCYCD3;3:GUS-GFP* lines crossed to *wox5-1* mutants and suggest that *WOX5* is required to exclude *CYCD3;3* expression from the QC.

To assess whether this downregulation is direct, we tested whether WOX5 binds the *CYCD3;3* promoter by using chromatin immunoprecipitation (ChIP)-quantitative PCR (qPCR) assays. Two of five regions tested from the *CYCD3;3* promoter, located 700 base pairs upstream of the transcriptional start site (TSS), and a fragment spanning the TSS showed 6.5-fold and 3.5-fold enrichment, respectively, compared to adjacent promoter regions (Figure 4B) and to *wox5-1* mutant controls (Figure 4C). In addition, no enrichment was seen for the *CYCD3;1* and the *CYCD6;1* promoter fragments, suggesting that WOX5 binding is not a conserved feature among *CYCD* promoters.

Taken together, we conclude that WOX5 binds the *CYCD3;3* promoter directly or indirectly, and it negatively regulates its expression in the QC.

Conclusions

Here, we demonstrate a new role for the transcription factor WOX5 in establishing and maintaining quiescence of the QC by regulating *CYCD3;3* activity. This establishes a direct link between core cell-cycle components and transcription factors in organizing the root stem cell niche. We observe that *CYCD1;1* and *CYCD3;3* stimulate cell division in the descendants of the hypophysis to form the embryonic columella. During embryogenesis, local action of WOX5 initiates quiescence in the QC, whose maintenance requires the continuing presence of WOX5. WOX5 acts in the QC through local suppression of *CYCD3;3* expression, and this function is needed to restrict cell division. Furthermore, an appropriate level of CYCD3;3 and CYCD1;1 is required for normal QC development. We conclude that tight control of *CYCD* activity by WOX5 is essential for QC quiescence and for the normal development of the QC. In contrast, in the nascent columella, WOX5 induces cell proliferation by acting on different targets and not primarily through *CYCD3;3* and *CYCD1;1*.

Hormone [22–25] and peptide [2] signaling pathways are known to regulate cell division in the QC, and recent advances have shown that a low proliferation rate of the QC is essential to maintain plant growth during stressful conditions [2, 3]. It remains to be shown how *WOX5* and *CYCD* presumably integrate these different signaling pathways to regulate cell division in the QC and maintain a functional stem cell niche during normal or stressed conditions.

Supplemental Information

Supplemental Information includes Supplemental Experimental Procedures, three figures, and one table and can be found with this article online at http://dx.doi.org/10.1016/j.cub.2014.07.019.

Acknowledgments

We thank Angela Marchbank and Joanne Kilby for excellent technical support. This project was supported by grants BB/E024858, BB/J009199/1, and BB/L009358/1 from the Biotechnology and Biological Sciences Research Council (BBSRC) to J.A.H.M. and W.D., by the ERA-NET in Plant Genomics (ERA-PG; project "Plant Stem Cell Network"; to J.A.H.M., W.D., and T.L.), by the EU-cofunded INTERREG IV Upper Rhine Project 692 A17 (to T.L.), by the Deutsche Forschungsgemeinschaft (SFB592), by the Excellence Initiative of the German federal and state governments (FRIAS; to T.L.), and by an EU Marie Curie Fellowship to E.A.

Received: May 1, 2014
Revised: June 9, 2014
Accepted: July 8, 2014
Published: August 7, 2014

References

1. van den Berg, C., Willemsen, V., Hendriks, G., Weisbeek, P., and Scheres, B. (1997). Short-range control of cell differentiation in the Arabidopsis root meristem. Nature *390*, 287–289.
2. Cruz-Ramírez, A., Díaz-Triviño, S., Wachsman, G., Du, Y., Arteága-Vázquez, M., Zhang, H., Benjamins, R., Blilou, I., Neef, A.B., Chandler, V., and Scheres, B. (2013). A SCARECROW-RETINOBLASTOMA protein network controls protective quiescence in the Arabidopsis root stem cell organizer. PLoS Biol. *11*, e1001724.
3. Heyman, J., Cools, T., Vandenbussche, F., Heyndrickx, K.S., Van Leene, J., Vercauteren, I., Vanderauwera, S., Vandepoele, K., De Jaeger, G., Van Der Straeten, D., and De Veylder, L. (2013). ERF115 controls root quiescent center cell division and stem cell replenishment. Science *342*, 860–863.
4. Sarkar, A.K., Luijten, M., Miyashima, S., Lenhard, M., Hashimoto, T., Nakajima, K., Scheres, B., Heidstra, R., and Laux, T. (2007). Conserved factors regulate signalling in Arabidopsis thaliana shoot and root stem cell organizers. Nature *446*, 811–814.
5. Dolan, L., Janmaat, K., Willemsen, V., Linstead, P., Poethig, S., Roberts, K., and Scheres, B. (1993). Cellular organisation of the Arabidopsis thaliana root. Development *119*, 71–84.
6. Aichinger, E., Kornet, N., Friedrich, T., and Laux, T. (2012). Plant stem cell niches. Annu. Rev. Plant Biol. *63*, 615–636.
7. Lau, S., Slane, D., Herud, O., Kong, J., and Jürgens, G. (2012). Early embryogenesis in flowering plants: setting up the basic body pattern. Annu. Rev. Plant Biol. *63*, 483–506.
8. Haecker, A., Gross-Hardt, R., Geiges, B., Sarkar, A., Breuninger, H., Herrmann, M., and Laux, T. (2004). Expression dynamics of WOX genes mark cell fate decisions during early embryonic patterning in Arabidopsis thaliana. Development *131*, 657–668.
9. Nieuwland, J., Scofield, S., and Murray, J.A. (2009). Control of division and differentiation of plant stem cells and their derivatives. Semin. Cell Dev. Biol. *20*, 1134–1142.
10. Boniotti, M.B., and Gutierrez, C. (2001). A cell-cycle-regulated kinase activity phosphorylates plant retinoblastoma protein and contains, in Arabidopsis, a CDKA/cyclin D complex. Plant J. *28*, 341–350.
11. Nakagami, H., Kawamura, K., Sugisaka, K., Sekine, M., and Shinmyo, A. (2002). Phosphorylation of retinoblastoma-related protein by the cyclin D/cyclin-dependent kinase complex is activated at the G1/S-phase transition in tobacco. Plant Cell *14*, 1847–1857.
12. Nowack, M.K., Harashima, H., Dissmeyer, N., Zhao, X., Bouyer, D., Weimer, A.K., De Winter, F., Yang, F., and Schnittger, A. (2012). Genetic framework of cyclin-dependent kinase function in Arabidopsis. Dev. Cell *22*, 1030–1040.
13. Wachsman, G., Heidstra, R., and Scheres, B. (2011). Distinct cell-autonomous functions of RETINOBLASTOMA-RELATED in Arabidopsis stem cells revealed by the Brother of Brainbow clonal analysis system. Plant Cell *23*, 2581–2591.
14. Wildwater, M., Campilho, A., Perez-Perez, J.M., Heidstra, R., Blilou, I., Korthout, H., Chatterjee, J., Mariconti, L., Gruissem, W., and Scheres, B. (2005). The RETINOBLASTOMA-RELATED gene regulates stem cell maintenance in Arabidopsis roots. Cell *123*, 1337–1349.
15. Menges, M., Pavesi, G., Morandini, P., Bögre, L., and Murray, J.A. (2007). Genomic organization and evolutionary conservation of plant D-type cyclins. Plant Physiol. *145*, 1558–1576.
16. Collins, C., Dewitte, W., and Murray, J.A. (2012). D-type cyclins control cell division and developmental rate during Arabidopsis seed development. J. Exp. Bot. *63*, 3571–3586.
17. Menges, M., Samland, A.K., Planchais, S., and Murray, J.A. (2006). The D-type cyclin CYCD3;1 is limiting for the G1-to-S-phase transition in Arabidopsis. Plant Cell *18*, 893–906.
18. Dewitte, W., Scofield, S., Alcasabas, A.A., Maughan, S.C., Menges, M., Braun, N., Collins, C., Nieuwland, J., Prinsen, E., Sundaresan, V., and Murray, J.A. (2007). Arabidopsis CYCD3 D-type cyclins link cell proliferation and endocycles and are rate-limiting for cytokinin responses. Proc. Natl. Acad. Sci. USA *104*, 14537–14542.
19. Riou-Khamlichi, C., Menges, M., Healy, J.M., and Murray, J.A. (2000). Sugar control of the plant cell cycle: differential regulation of Arabidopsis D-type cyclin gene expression. Mol. Cell. Biol. *20*, 4513–4521.
20. Sabatini, S., Heidstra, R., Wildwater, M., and Scheres, B. (2003). SCARECROW is involved in positioning the stem cell niche in the Arabidopsis root meristem. Genes Dev. *17*, 354–358.
21. ten Hove, C.A., Willemsen, V., de Vries, W.J., van Dijken, A., Scheres, B., and Heidstra, R. (2010). SCHIZORIZA encodes a nuclear factor regulating asymmetry of stem cell divisions in the Arabidopsis root. Curr. Biol. *20*, 452–457.
22. Ortega-Martínez, O., Pernas, M., Carol, R.J., and Dolan, L. (2007). Ethylene modulates stem cell division in the Arabidopsis thaliana root. Science *317*, 507–510.
23. Zhang, H., Han, W., De Smet, I., Talboys, P., Loya, R., Hassan, A., Rong, H., Jürgens, G., Paul Knox, J., and Wang, M.H. (2010). ABA promotes quiescence of the quiescent centre and suppresses stem cell differentiation in the Arabidopsis primary root meristem. Plant J. *64*, 764–774.
24. Zhang, W., Swarup, R., Bennett, M., Schaller, G.E., and Kieber, J.J. (2013). Cytokinin induces cell division in the quiescent center of the Arabidopsis root apical meristem. Curr. Biol. *23*, 1979–1989.
25. González-García, M.P., Vilarrasa-Blasi, J., Zhiponova, M., Divol, F., Mora-García, S., Russinova, E., and Caño-Delgado, A.I. (2011). Brassinosteroids control meristem size by promoting cell cycle progression in Arabidopsis roots. Development *138*, 849–859.

Current Biology, Volume *24*

Supplemental Information

WOX5 Suppresses *CYCLIN D* Activity to Establish Quiescence at the Center of the Root Stem Cell Niche

Celine Forzani, Ernst Aichinger, Emily Sornay, Viola Willemsen, Thomas Laux, Walter Dewitte, and James A.H. Murray

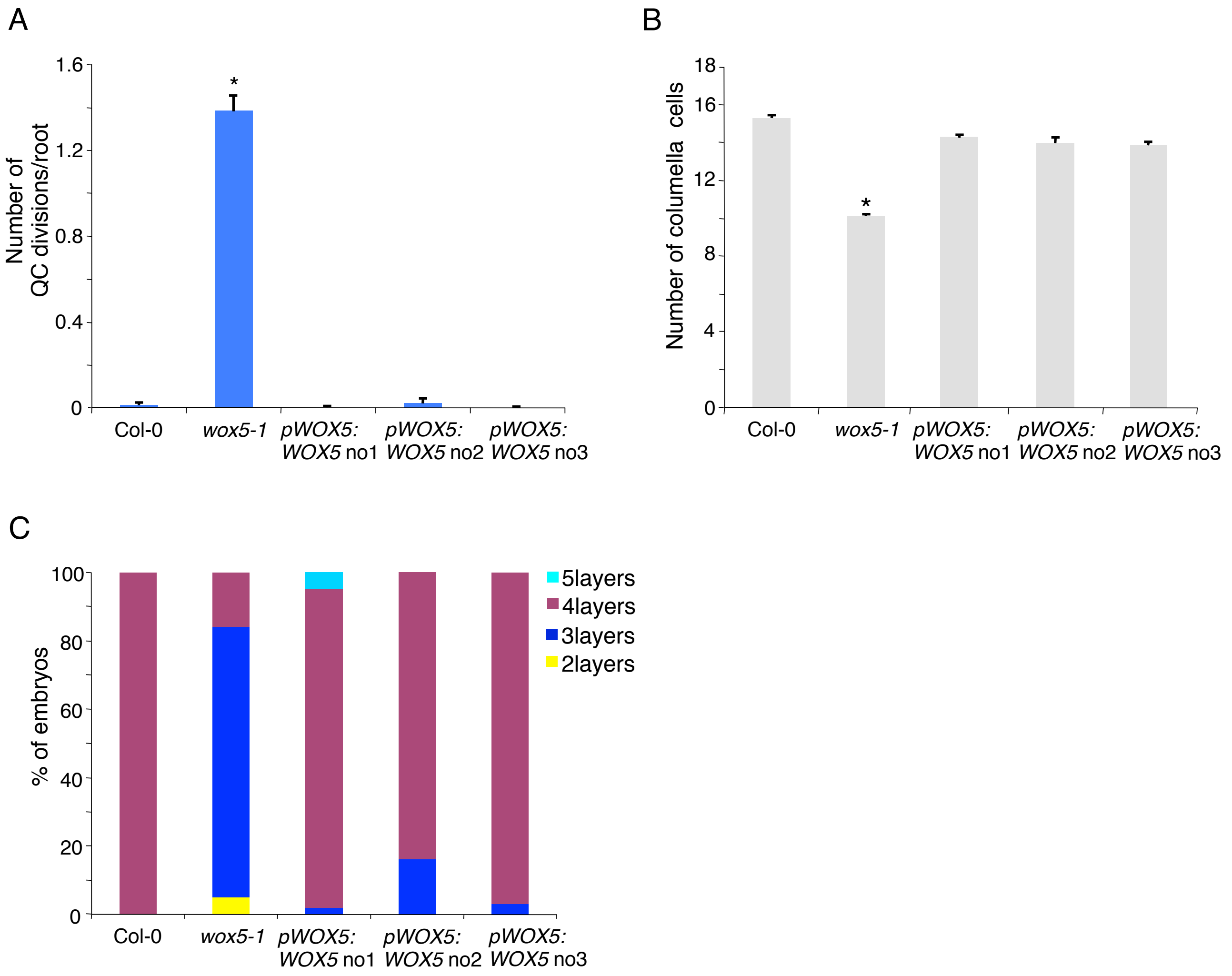

Figure S1. *wox5-1* complementation with a genomic WOX5 construct.

Related to figure 1.

Two additional *wox5-1* mutant plant lines expressing a WOX5 genomic DNA fragment under its own promoter *pWOX5:WOX5* no2 and no3 show rescue of *wox5-1* columella and QC defects, similar to the line *pWOX5:WOX5* no1 presented in Figure 1.

(A) Quantification of the number of transverse divisions occurring in the QC of mature embryos. (B) Quantification of the number of cells in the embryonic columella. (C) Quantification of the number of layers in the embryonic columella. (A-C) The number of embryos examined was: WT Col-0 (n=73), *wox5-1* (n=91), *pWOX5:WOX5* no1 (n=45), *pWOX5:WOX5* no2 (n=40), *pWOX5:WOX5* no3 (n=40). Error bars represent standard Errors. Student’s t test, *p<0.00001

QC: quiescent centre

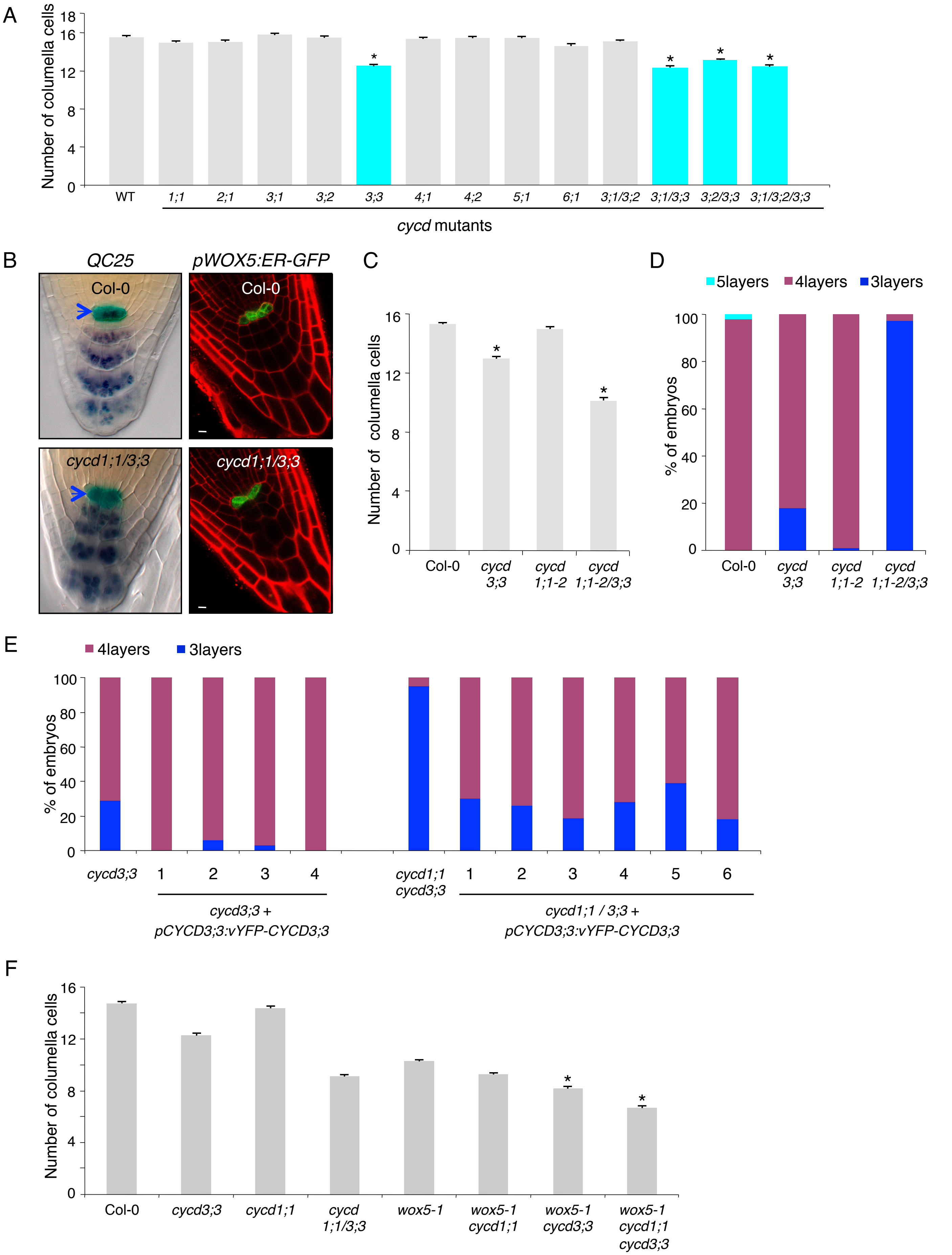
A
Number of columella cells
0
4
8
12
16
18
WT
1;1
2;1
3;1
3;2
3;3
4;1
4;2
5;1
6;1
3;1/3;2
3;1/3;3
3;2/3;3
3;1/3;2/3;3
cycd mutants
*
B
QC25
pWOX5:ER-GFP
Col-0
Col-0
cycd1;1/3;3
cycd1;1/3;3
C
Number of columella cells
0
4
8
12
16
Col-0
cycd 3;3
cycd 1;1-2
cycd 1;1-2/3;3
D
5layers
4layers
3layers
% of embryos
0
20
40
60
80
100
Col-0
cycd 3;3
cycd 1;1-2
cycd 1;1-2/3;3
E
4layers
3layers
% of embryos
0
20
40
60
80
100
cycd3;3
1
2
3
4
cycd3;3 + pCYCD3;3:vYFP-CYCD3;3
cycd1;1 cycd3;3
1
2
3
4
5
6
cycd1;1 / 3;3 + pCYCD3;3:vYFP-CYCD3;3
F
Number of columella cells
0
4
8
12
16
Col-0
cycd3;3
cycd1;1
cycd 1;1/3;3
wox5-1
wox5-1 cycd1;1
wox5-1 cycd3;3
wox5-1 cycd1;1 cycd3;3

Figure S2. *CYCD3;3*, *CYCD1;1* and *WOX5* regulate cell division in the embryonic columella.

Related to figure2.

(A) Quantification of the number of columella cells in the mature embryo. Approximately 30 embryos were counted for each genotype. *cycd3;3* mutants show less columella cells in the embryonic root compared to the WT Col-0 or to other *cycd* mutants. (B) *QC25* and *pWOX5:ER-GFP* expression in WT Col-0 and *cycd1;1 cycd3;3* 5-day-old seedlings. The enhancer trap line *QC25* expresses the ß-glucuronidase (GUS activity indicated by the blue arrow) in the QC. Starch granules in amyloplasts were stained dark blue by lugol (left panel). Cell walls were counterstained by propidium iodide (right panel). Scale bars= 5 μm. (C-D) A second loss-of-function allele of *CYCD1;1, cycd1;1-2* in combination with *cycd3;3* recapitulates the phenotypic abnormalities observed in *cycd1;1 cycd3;3* mutant plants. Approximately 50 embryos were counted for each genotype. (C) Quantification of the number of columella cells in the mature embryo. (D) Quantification of the number of layers in the embryonic columella. (E) A CYCD3;3 genomic DNA fragment tagged with vYFP was expressed under the control of the *CYCD3;3* promoter in *cycd3;3* or *cycd1;1 cycd3;3* mutants. Four different transgenic lines show complementation of the *cycd3;3* mutant defects. Six different transgenic lines show complementation of the *cycd1;1 cycd3;3* mutant defects. Quantification of the number of layers in the embryonic columella. Approximately 30 embryos were counted for each genotype. (F) A reduced number of columella cells was observed in *wox5 cycd1;1 cycd3;3* embryos compared to *wox5-1* or *cycd1;1 cycd3;3* embryos. Quantification of the number of columella cells in the mature embryo. The number of embryos examined was WT Col-0 (n=108), *cycd3;3* (n=101), *cycd1;1* (n=102), *cycd1;1 cycd3;3* (n=119), *wox5-1* (n=107), *wox5 cycd1;1* (n=118), *wox5 cycd3;3* (n=109), *wox5 cycd1;1 cycd3;3* (n=116).

(A, C and F) Error bars represent standard errors. Student t test *<0.00001. (F) * indicates a statistical difference between *cycd1;1 cycd3;3* and the different mutant combinations.

QC: quiescent centre

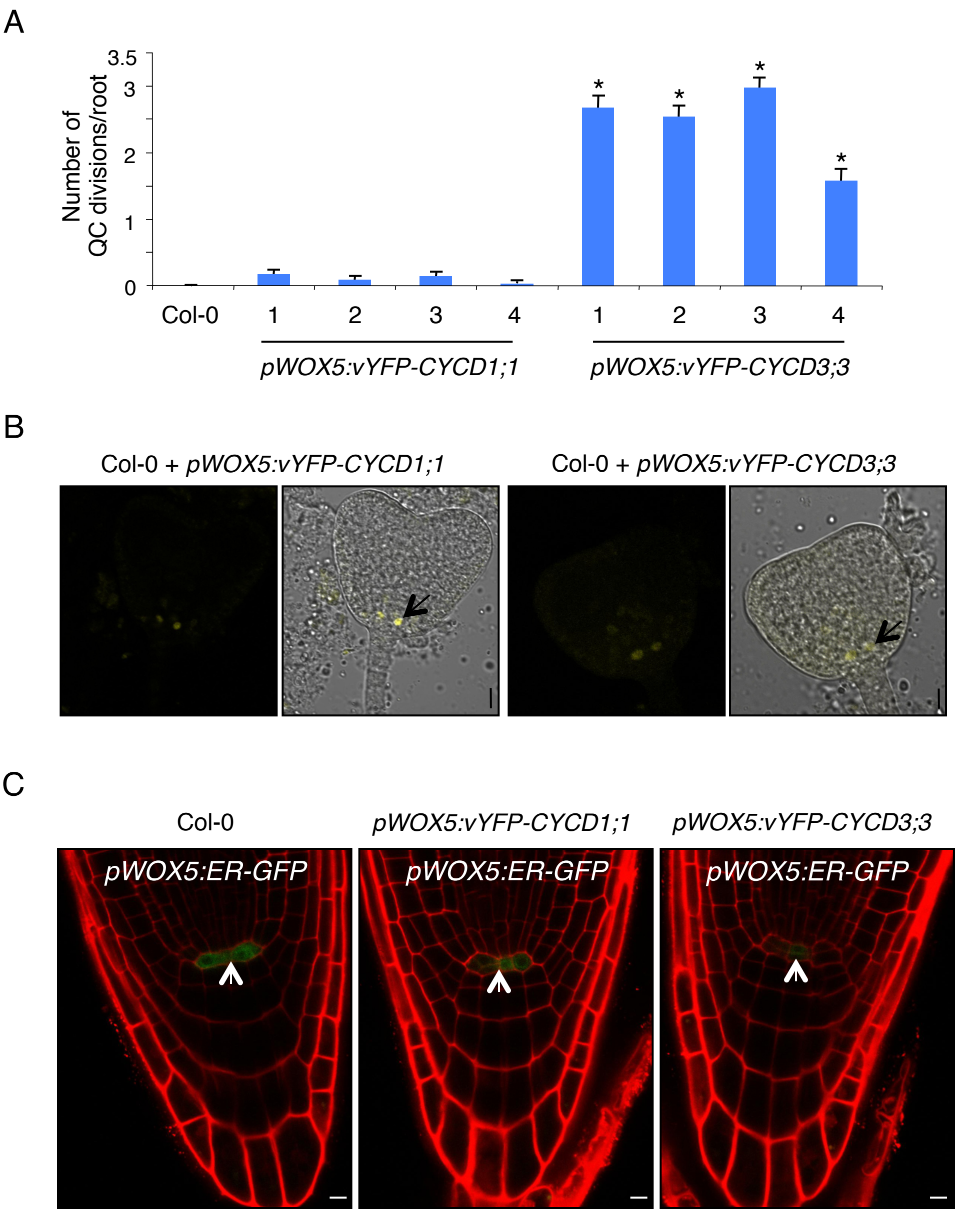

Figure S3. Increasing *CYCD* expression in the QC is sufficient to induce cell division in the QC.

Related to figure 3.

(A-C) A CYCD1;1 or a CYCD3;3 genomic DNA fragment tagged with vYF*P* was expressed under the control of the *WOX5* promoter in WT Col-0 plants. (A) Quantification of the number of transverse divisions occurring in the QC of mature embryos. Approximately 30 embryos were counted for each genotype. Error bars represent standard errors. Student t test *<0.00001 (B) The subcellular localization of *pWOX5:vYFP-CYCD1;1* or *pWOX5:vYFP-CYCD3;3* fusion proteins was examined in WT Col-0 heart stage embryos. The CYCD fusion proteins were detected in the nuclei (indicated by the black arrow).

The left panel shows the vYFP fluorescence and the right panel the vYFP fluorescence merged with the differential interference contrast image (DIC). Scale bars, 10 *μ*m. (C) Five-day-old seedlings showing *pWOX5:ER-GFP* expression in the QC of WT Col-0 and *pWOX5:vYFP-CYCD1;1* or *pWOX5:vYFP-CYCD3;3* expressed in WT Col-0. Cell walls were counterstained by propidium iodide. F1 populations were used with *pWOX5:ER-GFP* being hemizygous in all the genotypes. White arrows indicate the QC. Scale bars= 5 *μ*m.

Supplemental Table

Table S1. Quantification of the number of CSC layers in *cycds* and *wox5* mutant combinations.

Related to figure 2.

	**0 CSC (%)**	**1 CSC (%)**	**2 CSC (%)**	**N**
Col-0	0	84	16	31
cycd3;3	6	94	0	31
cycd1;1	3	58	39	31
cycd1;1 cycd3;3	32	68	0	32
wox5-1	100	0	0	31
wox5-1 cycd1;1	97	3	0	32
wox5-1 cycd3;3	100	0	0	31
wox5-1 cycd1;1 cycd3;3	94	6	0	32

3-day-old seedlings were visualized by propidium iodide. The percentage of roots with 0, 1 or 2 layers of CSC were counted. The graph in figure 2O scored these seedlings for their number of transverse divisions occurring in the QC.

Supplemental Experimental procedures

Plant growth conditions

Seeds were germinated on root medium (2.2 g L^{-1} Murashige Skoog, 0.75 % sucrose and 1.5 % agar pH 5.8) or GM medium (4.4 g L^{-1} Murashige Skoog, 1.5 % sucrose, 0.5 g L^{-1} 2-(N-Morpholino) ethanesulfonic acid sodium salt (MES) and 1 % agar, pH 5.8). The seeds were stratified at 4°C for 48 h and were then transferred to 22°C under long day conditions (16 h light, 8 h dark) for germination and growth.

Plant lines and constructs

The *Arabidopsis thaliana* ecotype Columbia 0 was used for all the experiments. The *cycd3;1*, *cycd3;2*, *cycd3;3* [S1], *cycd1;1* [S2], *wox5-1*, *wox5-3* [S3] mutants, *QC25, QC46* [S4] and *pWOX5:ER-GFP* [S5] lines have been described previously. The loss-of-function mutant line *cycd1;1-2* (SM_3.20203) was obtained from the Nottingham Arabidopsis stock centre. It has a T-DNA insertion in the 2nd intron of the *CYCD1;1* gene. The site of insertion sequenced 3' from the T-DNA is: ATGATTATATTAATCAGCTTTAATTTCTGTAAAATAGGAAACGAGTGGTTG GCCAATGCAACTTTT. The primers used for genotying are: T-DNA: ACCGTCGACTACCTTTTTCTTGTAGTG, CYCD1;1 forward: CTTTACGCTCGTCGATTACCG and CTCD1;1 reverse: GCATTGGCCAACCACTCGTTTC. The absence of the *CYCD1;1* transcript in this line was confirmed by RT-PCR with primers spanning the T-DNA (data not shown). Plants were transformed using the floral dipping method [S6]. Primary transformants were selected on GM medium containing kanamycin (50 µg/ml) or DL-phosphinothricin (12 µg/ml).

The *CYCD1;1* and *CYCD3;3* promoter sequence was amplified by PCR from total genomic DNA derived from Col-0 seedlings. *pCYCD1;1* (5665 bp upstream of the transcriptional start site (TSS)) and *pCYCD3;3* (4805 bp upstream of the TSS) were introduced into the gateway vector pENTR/D-TOPO (Invitrogen) with a SmaI site introduced at the 5' end of the promoter. These constructs were then recombined into the binary vector pKGWFS7 [S7] generating *pCYCD1;1:eGFP-GUS* and

pCYCD3;3:eGFP-GUS.

pWOX5:vYFP-CYCD1;1, *pWOX5:vYFP-CYCD3;3* and *pCYCD3;3:vYFP-CYCD3;3* constructs were expressed in plants from the binary vector pGreenII 0229 [S8]. The pGreenII 0229 vector was modified to be able to make these constructs. First, the *35S* promoter was cloned ApaI/XhoI into the pGreenII 0229. Additional restriction sites in the *p35S* primers created new restriction sites in the pGreenII 0229 vector: ApaI-PmeI/*p35S*/MluI-XhoI. The vYFP tag was cloned behind the *35S* promoter MluI/XhoI. Additional restriction sites in the vYFP primers created: MluI/vYFP/NotI-AscI-XhoI. The *CYCD3;3* (4805 bp upstream of the TSS) or the *WOX5* (4658 bp upstream of the TSS) promoters were cloned SmaI/AscI into this modified pGreenII 0229 by replacing the *35S* promoter PmeI/MluI. The genomic DNA sequence of CYCD1;1 or of CYCD3;3 including the 3' UTR was amplified by PCR from total genomic DNA derived from Col-0 seedlings. Each fragment was cloned in frame to the vYFP N-terminal tag NotI/AscI behind the *WOX5* promoter. In addition, the CYCD3;3 gDNA fragment was cloned in frame to the vYFP N-terminal tag NotI/AscI behind the *CYCD3;3* promoter.

The *pWOX5:WOX5* construct was expressed in the *wox5 QC184* mutant background from the binary vector pGreenII. A WOX5 genomic fragment was amplified using the primers: CCCGGGTGGTAGCGAACTAGGAATTGTATGTGC and GGCGCGCCTCCGACGGAACTTGAGTTTGCTTC and cloned AscI/XmaI into pGreenII.

Histochemical staining

5-day-old seedlings were fixed in 90 % acetone for 30 min at 4°C, washed three times with 50 mM sodium phosphate buffer (pH 7.0) and subsequently stained for up to 16 hours at 37°C, in 50 mM sodium phosphate buffer (pH 7.0), 10 mM $K_3Fe(CN_6)$, 10 mM $K_4Fe(CN_6)$ containing 1 mM 5-bromo-4-chloro-3-indolyl-ß-D-glucuronide. Subsequently, seedlings were incubated in Lugol's solution (SIGMA) for 30 seconds and rinsed with water. Root tips were mounted in chloral hydrate (2.7 g chloral hydrate in 1 ml water) and were visualized with a Leica MZ16FA stereomicroscope using differential interference contrast optics.

Confocal Microscopy

Dry seeds were imbibed in water O/N. Mature embryos were popped out from the seed and stained in fixative (50 % ethanol / 10 % acetic acid) for 24 h. Embryos were rinsed with water and incubated in 1 % periodic acid for 40 min. at room temperature. Embryos were rinsed again and then stained with a Schiff reagent (100 mM sodium metabisulphite, 0.15N HCL, propidium iodide 100 µg/ml) for 1 h at room temperature. Embryos were rinsed with water and cleared with a chloral hydrate solution (2.7 g chloral hydrate in 1 ml water). Embryos were then mounted on a slide. Roots from 5-day-old seedlings were directly mounted in propidium iodide solution (40 µg/ml) to stain the cell wall. Samples were visualized by confocal microscopy Zeiss LSM 710.

Chromatin Immunopreciptiation

The ChIP was carried out using an anti-WOX5 antibody to co-immunoprecipitate the DNA interacting with a WOX5-GR fusion protein expressed from the *35S* promoter in the WT Col-0 background. Roots of 7-day-old seedlings, treated with 5 µM dexamethasone and 0.15 % silwet were used for ChIP experiments according to Kwon *et al.* [S9] with some minor modifications. 1 to 1.2 g of root tissue was used per replicate. Lysed nuclei were sonicated with a Bioruptor sonicator (Diagenode) to a fragment size of 300 to 600 bp. Pre-clearing was performed with a 1:1 mixture of Protein-A/Protein-G dynabeads (Invitrogen) for 1.5 h. The DNA was precipitated by using an anti-WOX5 antibody at 4°C for 16 hours followed by incubation with a 1:1 mixture of Protein-A/Protein-G dynabeads for 2 hours. Cross-linking was reversed by incubation of the beads in 10 % chelex at 95°C for 10 min, followed by incubation with 20 µg proteinase K at 50°C for 30 min and at 95°C for 10 min. The supernatant was collected and combined with one washing step of the chelex/dynabeads with TE buffer. 3 µl per well was used for qPCR using SYBR Green II (Roche).

List of primers used to amplify the different promoter regions:

CYCD3;3-1750:

CTAATGGAACATATTGTAGACCTATTGG

AACAAAGAAATTCCTTATCATATCGCT

CYCD3;3 -700:

ACACAATGACAGTGACTCTACACATTACG

CGTCATTAGTACTTTGATTTTGGTTAATACAAC

CYCD3;3_5UTR

AGAGGACAAGCGTGAAATAAAACCCT

CTCTGGATTCTTCACTCTGTGTGAGA

CYCD3;3_intron

GATCTCTTGAATCTACTTGATTTTGTTGT

GGCTAACACAGAAGGACTAAAACTCAG

CYCD3;3_3UTR

TGTCAACAACCCATCAAAATGTGT

TCCATAAACACAAGAACCGAGAACCA

CYCD3;1-400

TTGTGTGTTCATCATCCTCTTGAG

GCCGATAAGTTCCGTTTCTTAAAG

CYCD6;1-1000

TAACTGTTTGGACGAGATTCCAAAGT

GTTGTCATTTACTCATAAATAGAGCTGATG

CYCD6;1-100

ACCATCTCTCTCTATCTCACAATAAAGAC

TTCTTGAATTGGTGTTAGAAGATGAAGA

eIF4

CACGCCCTGGAGTTCCAACAAC

GCAAATTGAGAAGGTCATGAGG

Supplemental References

S1. Dewitte, W., Scofield, S., Alcasabas, A.A., Maughan, S.C., Menges, M., Braun, N., Collins, C., Nieuwland, J., Prinsen, E., Sundaresan, V., *et al*. (2007). Arabidopsis CYCD3 D-type cyclins link cell proliferation and endocycles and are rate-limiting for cytokinin responses. Proc. Natl. Acad.

Sci. USA *104*, 14537-14542.

S2. Masubelele, N.H., Dewitte, W., Menges, M., Maughan, S., Collins, C., Huntley, R., Nieuwland, J., Scofield, S., and Murray, J.A. (2005). D-type cyclins activate division in the root apex to promote seed germination in Arabidopsis. Proc. Natl. Acad. Sci. USA *102*, 15694-15699.

S3. Sarkar, A.K., Luijten, M., Miyashima, S., Lenhard, M., Hashimoto, T., Nakajima, K., Scheres, B., Heidstra, R., and Laux, T. (2007). Conserved factors regulate signalling in Arabidopsis thaliana shoot and root stem cell organizers. Nature *446*, 811-814.

S4. Sabatini, S., Heidstra, R., Wildwater, M., and Scheres, B. (2003). SCARECROW is involved in positioning the stem cell niche in the Arabidopsis root meristem. Genes Dev. *17*, 354-358.

S5. ten Hove, C.A., Willemsen, V., de Vries, W.J., van Dijken, A., Scheres, B., and Heidstra, R. (2010). SCHIZORIZA encodes a nuclear factor regulating asymmetry of stem cell divisions in the Arabidopsis root. Curr. Biol. *20*, 452-457.

S6. Clough, S.J., and Bent, A.F. (1998). Floral dip: a simplified method for Agrobacterium-mediated transformation of Arabidopsis thaliana. Plant J. *16*, 735-743.

S7. Karimi, M., Inze, D., and Depicker, A. (2002). GATEWAY vectors for Agrobacterium-mediated plant transformation. Trends Plant Sci. *7*, 193-195.

S8. Hellens, R.P., Edwards, E.A., Leyland, N.R., Bean, S., and Mullineaux, P.M. (2000). pGreen: a versatile and flexible binary Ti vector for Agrobacterium-mediated plant transformation. Plant Mol. Biol. *42*, 819-832.

S9. Kwon, C.S., Chen, C., and Wagner, D. (2005). WUSCHEL is a primary target for transcriptional regulation by SPLAYED in dynamic control of stem cell fate in Arabidopsis. Genes Dev. *19*, 992-1003.
